# Identification of polymer surface adsorbed proteins implicated in pluripotent human embryonic stem cell expansion†
†Electronic supplementary information (ESI) available. See DOI: 10.1039/c6bm00214e



**DOI:** 10.1039/c6bm00214e

**Published:** 2016-07-28

**Authors:** Moamen Hammad, Wei Rao, James G. W. Smith, Daniel G. Anderson, Robert Langer, Lorraine E. Young, David A. Barrett, Martyn C. Davies, Chris Denning, Morgan R. Alexander

**Affiliations:** a Laboratory of Biophysics and Surface Analysis , School of Pharmacy , University of Nottingham , Nottingham , NG7 2RD , UK . Email: morgan.alexander.nottingham.ac.uk; b Centre for Analytical Bioscience , School of Pharmacy , University of Nottingham , Nottingham , NG7 2RD , UK; c Wolfson Centre for Stem Cells , Tissue Engineering and Modelling (STEM) , University of Nottingham , Nottingham NG7 2RD , UK; d David H. Koch Institute for Integrative Cancer Research , Massachusetts Institute of Technology , Cambridge , MA 02139 , USA

## Abstract

Improved biomaterials are required for application in regenerative medicine, biosensing, and as medical devices. The response of cells to the chemistry of polymers cultured in media is generally regarded as being dominated by proteins adsorbed to the surface. Here we use mass spectrometry to identify proteins adsorbed from a complex mouse embryonic fibroblast (MEF) conditioned medium found to support pluripotent human embryonic stem cell (hESC) expansion on a plasma etched tissue culture polystyrene surface. A total of 71 proteins were identified, of which 14 uniquely correlated with the surface on which pluripotent stem cell expansion was achieved. We have developed a microarray combinatorial protein spotting approach to test the potential of these 14 proteins to support expansion of a hESC cell line (HUES-7) and a human induced pluripotent stem cell line (ReBl-PAT) on a novel polymer (*N*-(4-Hydroxyphenyl) methacrylamide). These proteins were spotted to form a primary array yielding several protein mixture ‘hits’ that enhanced cell attachment to the polymer. A second array was generated to test the function of a refined set of protein mixtures. We found that a combination of heat shock protein 90 and heat shock protein-1 encourage elevated adherence of pluripotent stem cells at a level comparable to fibronectin pre-treatment.

## Abstract 2

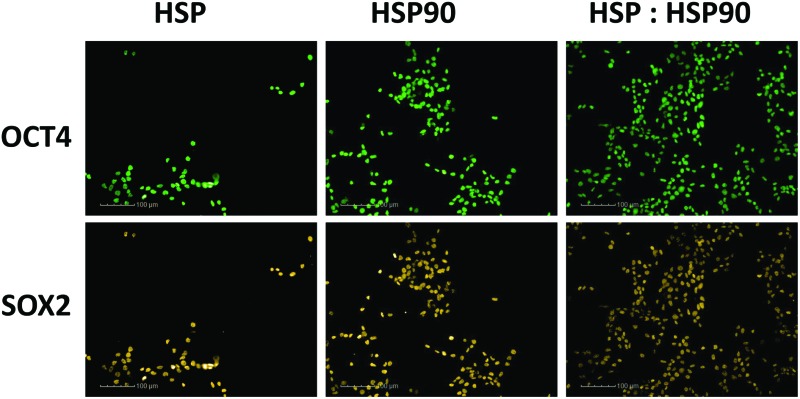
The discovery of heat shock proteins as candidates for human pluripotent stem cell culture using high throughput screening.

## Introduction

Human embryonic stem cells (hESC) are pluripotent cells derived from the inner cell mass (ICM) of a developing blastocyst embryo and have the capacity to differentiate into many cell types within the body.^4^ Pluripotent cells offer a promising source of replacement cells for patients to treat a range of conditions in regenerative medicine. In practice many hurdles have to be overcome before the technology is robust enough to be accepted as a routine therapy. For the clinical setting, this includes the removal of biologically-derived components often used for hESC culture, such as mouse embryonic fibroblast conditioned medium (MEF-CM)^5^ or undefined surfaces, such as Matrigel.^6,7^ Currently many labs have achieved a defined culture environment using E8 defined medium and the use of chemical substrates (*i.e.* Synthemax and Stemadhere).^8–10^ However it is unclear which proteins are involved in regulating stem cell interactions and how these proteins maintain pluripotency when adsorbed on a substrate. Proteins have been identified within MEF-CM that maintain hESC pluripotency and are involved in cell signalling, cell–cell interaction, and cell adhesion.^11–13^

Studies in recent years have focused on the proteomic analysis of the secretome of MEF-CM.^14^ However a detailed proteomic study of the proteins that are retained by the surfaces in contact with MEF-CM has not been attempted previously, with a number of studies focusing only on the content of bovine serum albumin (BSA) and related proteins on the surface.^15,16^ We used proteomics to identify proteins adsorbed to a plasma etched tissue culture polystyrene (PE-TCPS) surface from MEF-CM. Since PE-TCPS surfaces have been shown to be a well-defined, robust system for pluripotent hESC proliferation,^1^ we used this surface as a model for the systematic elucidation of the proteins adsorbed from MEF-CM that correlated with pluripotent expansion. We identified strongly bound proteins, released from the surface using vigorous rinsing and identified by a combination of gel electrophoresis and liquid chromatography mass spectrometry (LC-MS). To explore the utility of these proteins we printed them as *protein microarrays* on a novel polymer which is a promising candidate for stem cell expansion: poly(*N*-(4-hydroxyphenyl) methacrylamide) as it has shown HUES-7 hESC adherence and expansion at levels comparable with PE-TCPS but using a media (StemPro) with much reduced complexity compared to MEF-CM.^2^

We demonstrate here a methodology for testing protein pre-treatments to determine their significance in hESC culture which yields some interesting molecules implicated in pluripotent expansion of stem cells. We illustrate that while this method can be used on a single surface identity presented as spots, it is also amenable to printing proteins identified to be important on arrays of different polymers presented as spots in order to explore and investigate their potential as adsorbates to control hESC response.

## Results

### Proteomics

For comparison, PE-TCPS and TCPS Nuclon Delta 4-well plates were incubated with 5 mL MEF-CM per well at 37 °C for 1 hour prior to extraction of the surface-bound proteins. PE-TCPS is known to be supportive for hESC culture whereas TCPS is not; the proteins adsorbed to each surface were identified by gel electrophoresis followed by LC-MS of the main bands. A total of 71 unique proteins were detected and identified from the proteomics analysis of murine and bovine proteins adsorbed to PE-TCPS and TCPS (Table 1). Most of the proteins were mouse in origin, suggesting they emanated from the MEF-CM. However 28 of the proteins were detected to have come from bovine sources. These bovine proteins are most likely to originate from the BSA employed in the MEF-CM.

**Table 1:** Proteins adsorbed to two different types of surfaces, TCPS and PE-TCPS from MEF-CM (murine proteins, in normal font) and BSA (bovine proteins, in italics)

TCPS unique proteins	Shared	PE-TCPS unique proteins
Slit2 protein6	Tenascin	Fibronectin
Slit homolog 3	Myosin-9	Desmoplakin
Immunoglobulin superfamily member 10	Basement membrane-specific heparan sulfate proteoglycan core protein	Heat shock protein 1-like
Proenkephalin-A	Enolase 1, alpha non-neuron	Glyceraldehyde-3-phosphate dehydrogenase
Protein Col6a3	Collagen alpha-1(XVIII) chain	Reticulon
Sushi, nidogen and EGF-like domain-containing protein 1	Procollagen C-endopeptidase enhancer 1	Mini-agrin
Matrilin-2	Lysyl oxidase homolog 1	Ubiqutin subunit 1
Thrombospondin-1	Adipocyte enhancer-binding protein 1	Heat shock protein 90, beta (Grp94), member 1
Protein-lysine 6-oxidase	Angiopoietin-related protein 4	*Platelet factor 4*
Gremlin 2 homolog, cysteine knot superfamily	Vimentin	*Tetranectin*
Bone morphogenetic protein 1	Albumin 1	*Serum Amyloid P-component*
Fibrillin-1	Matrix Gla protein	*Alpha-2-antiplasmin*
Pyruvate kinase PKM	Nascent polypeptide-associated complex subunit alpha	*Beta-lactoglobulin*
60S ribosomal protein L12	Latent-transforming growth factor beta-binding protein 1	*Ovarian and testicular apolipoprotein N*
Inhibin beta B chain	Serine (Or cysteine) peptidase inhibitor, clade E, member 2, isoform CRA_b	
Periostin	*Serum albumin*	
Plasminogen activator, urokinase	*Apolipoprotein A-I*	
Transforming growth factor, beta induced	*Apolipoprotein A-IV*	
Latent-transforming growth factor beta-binding protein 2	*Alpha-2-macroglobulin*	
Latent transforming growth factor beta binding protein 4	*Antithrombin-III*	
*Serotransferrin*	*Complement C3*	
*Complement C4*	*Endopin 2B*	
*Lipopolysaccharide-binding protein*	*Alpha-1-antiproteinase*	
*Gelsolin*	*Serpin A3-1*	
*Vitamin D-binding protein*	*Serpin A3-3*	
*Inter-alpha inhibitor H4*	*Serpin A3-6*	
	*Serpin A3-7*	
	*Beta-2-glycoprotein 1*	
	*Hemoglobin subunit alpha-1*	
	*Hemoglobin subunit beta-A*	
	*Kappa-casein*	

A total of 14 proteins were found to uniquely adsorb to PE-TCPS, with 8 and 6 proteins of mouse and bovine origin respectively. We focused on proteins unique to the PE-TCPS surface as we hypothesised that these may be responsible for the ability of the PE-TCPS surface (and not TCPS) to be able to support long term hESC culture using MEF conditioned media. It appears that the PE-TCPS surface has a reduced total number of proteins upon media exposure compared to the TCPS surface, but those that are adsorbed are hypothesised to create a suitable environment for the maintenance of pluripotency in hESCs. To test this hypothesis the identified proteins were obtained from commercial suppliers: human fibronectin (FN), human agrin (MA), human glyceraldehyde-3-phosphate dehydrogenase (GAPDH), human reticulon 4 interacting protein 1 (RTU), human desmoplakin I + II peptide (DPK), human ubiquitin (UQ), human heat shock protein 1-like (HSP), human heat shock protein 90 (HSP90), human platelet factor 4 (PF4), human tetranectin (TN), human serum amyloid P-component (SAP), human alpha-2-antiplasmin (A2A), and bovine beta-lactoglobulin (BL) (ESI Table 1†). Although we identified bovine ovarian and testicular apolipoprotein as a protein which uniquely adsorbed to PE-TCPS, we were unable to find a supplier from which to source it, thus only 13 of the 14 proteins identified by the proteomics were taken forward for detailed investigation. Human proteins were used where possible with the exception of beta-lactoglobulin (bovine). Human desmoplakin I + II peptide was also used instead of desmoplakin. Reticulon 4 interacting protein 1 was used in place of reticulon 4 protein. Mini-agrin is a murine protein and therefore human agrin was used as an alternative. Heat shock protein 90 was also used in place of *heat shock protein 90 beta member 1*. The proteomics analysis also identified ubiquitin subunit 1, but the full length ubiquitin was used.

### Primary screening of protein-material microarrays

**Fig. 1:**
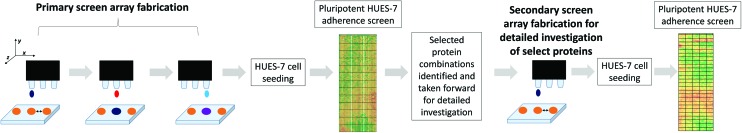
Piezo printing of biomolecules on the same *x*, *y* coordinates as the polymer spots (in orange). From left to right: protein spotting onto a polymer microarray, followed by mixing with another protein solution. For the primary screen, proteins were mixed pairwise at 70/30% at 0.1, 0.5, and 1 fmol. Proteins were kept in solution and prevented from drying out by using low temperature and high humidity conditions and by piezo dispensing of water. After printing the slide was kept in cold humid conditions for 6 hours. Afterwards the array was seeded with HUES-7 cells at a density of 1 × 10^6^ cells for 24 hours. OCT-4 immunocytochemistry staining was carried out to quantify the number of cells per spot; all results presented here refer to the pluripotent cell population per spot. A secondary microarray screen for more detailed investigation was generated from ‘hit’ protein combinations supporting pluripotent HUES-7 cell adherence were further investigated and mixed pairwise at 30, 50, and 70% at 0.1, 0.5, 1, 2 and 4 fmol.

High throughput screening of polymer libraries has identified a novel material ((*N*-(4-hydroxyphenyl) methacrylamide), polyHPhMA) which supports hESC culture at a level comparable with PE-TCPS in MEF-CM but using a far simpler commercial media-StemPro.^2,3^ It was hypothesised that the proteins which uniquely adsorbed to the PE-TCPS may also support hESC culture on other surfaces, and in particular we tested them on polyHPhMA. A microarray composed of polyHPhMA spots was generated by on-slide polymerisation onto a base poly(hydroxylethyl methacrylate) (pHEMA) coated glass slide. Using a robotic piezo spotter, nanolitre quantities of proteins were printed on individual polymer spots; a schematic of the methods is shown in Fig. 1.

**Fig. 2:**
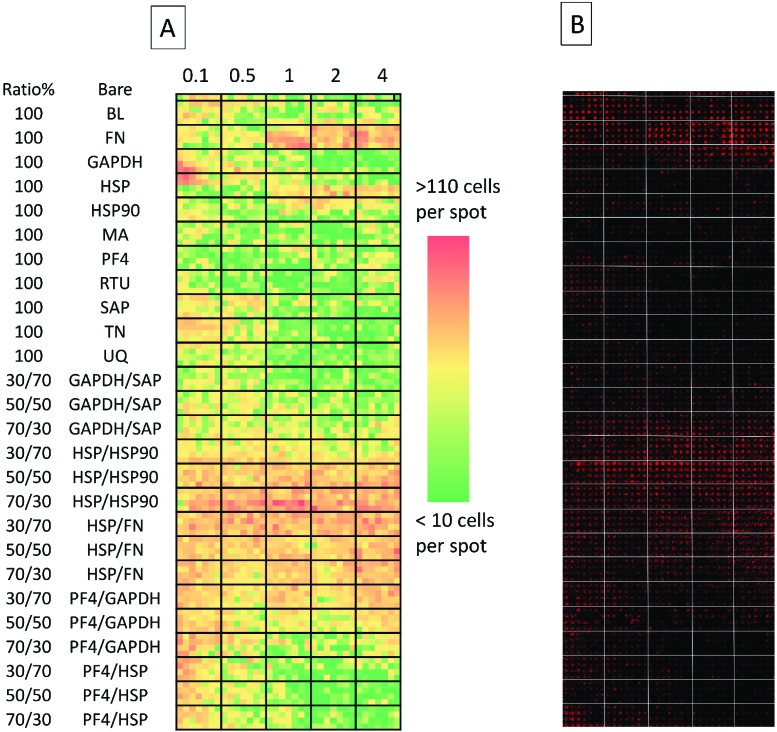
(A) Collated images of secondary screen. From left to right: heat map representative of cell attachment is shown (red signifies high number of cells, green signifies a low number of cells). Top row depicts cell adherence on non-spotted *N*-(4-hydroxyphenyl) methacrylamide surfaces. Columns are separated by bold black lines; from left to right: the first columns depicts cell adherence on surfaces spotted with 0.1 fmol, followed by cell adherence on 0.5, 1, 2 and 4 fmol. Rows (separated by horizontal black lines) represent either different proteins spotted on the surface or different co-adsorbed ratios of the same proteins. Each ‘block’ within the emboldened black borders depict replicates which are 7 spots across and 4 spots down (making for 28 replicates) and (B) detection of Oct-4 expressing cells on the array using Cy3 labelled antibody, each spot acquired separately and stitched together). BL = Beta-lactoglobulin, TN = tetranectin, PF4 = platelet factor 4, GAPDH = glyceraldehyde-3-phosphate dehydrogenase, MA = agrin, UQ = ubiquitin, HSP90 = heat shock protein 90, HSP = heat shock protein-1-like, SAP = serum amyloid P, FN = fibronectin and RTU = reticulon 4 interacting protein 1.

The proteins identified in the proteomics study (Table 1) were spotted combinatorially on polyHPhMA, mixing thirteen proteins pairwise (30/70) resulting in 169 combinations (with seven replicates for each combination); these were initially screened at 0.1, 0.5, and 1 fmol concentrations to investigate how the protein concentration could affect cell adherence (array lay-out on ESI Table 2†). To implement this *primary screen*, the human embryonic cell (hESC) line, HUES-7, was used. HUES-7 cells were seeded on the array at a density of 1 × 10^6^ cells in StemPro medium with 1% w/v antibiotic (penicillin/streptomycin) and cultured for 24 hours in a 4 well plate. Uniform cell seeding distribution was achieved by thorough mixing of the cell suspension in excess volume prior to seeding cells on a level surface, followed by minimal handling of the arrays during culture. Previous work has shown that this procedure results in pluripotent HUES-7 cell adherence on polymer microarrays which correlates with the surface chemistry of the arrayed polymers.^3^ OCT-4 immunocytochemistry staining was carried out to quantify the number of pluripotent cells per spot; all results presented here refer to the pluripotent cell population per spot. The control used was a non-pretreated polymer surface. Statistical significance was obtained using the unpaired *t*-test method and corrected using the Bonferroni correction method and found to be at *p* < 0.0001 for the primary screen.

From the primary screen we determined 76 protein adsorption combinations which supported greater HUES-7 cell adherence than the non-pretreated polymer spots (*p* < 0.0001, ESI Fig. 3 and 4†). Adsorption of GAPDH, HSP, HSP90, MA, PF4, RTU, SAP, TN and UQ in both pure and in combination supported cell adherence, and were thus taken forward for investigation in a *second generation screen* to investigate a larger number of combinations.

### Secondary protein-material screen

**Fig. 3:**
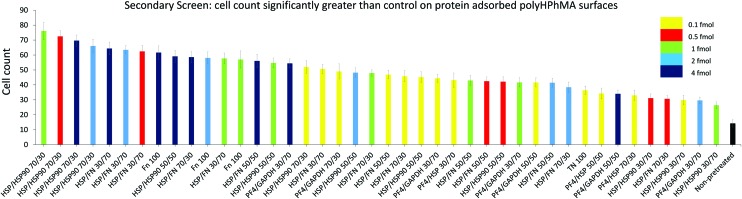
HUES7 cell adherence to samples from secondary screen, *n* = 28 from one microarray, raw data in Fig. 2B. Bars are colour coded to represent concentration of spotted proteins. Yellow: 0.1 fmol, red: 0.5 fmol, green: 1 fmol, cyan: 2 fmol, and dark blue: 4 fmol. The non-pretreated surface is shown in black. Error bars are standard error of the mean. BL = Beta-lactoglobulin, TN = tetranectin, PF4 = platelet factor 4, GAPDH = glyceraldehyde-3-phosphate dehydrogenase, MA = agrin, UQ = ubiquitin, HSP90 = heat shock protein 90, HSP = heat shock protein-1-like, SAP = serum amyloid P, FN = fibronectin and RTU = reticulon 4 interacting protein 1.

**Fig. 4:**
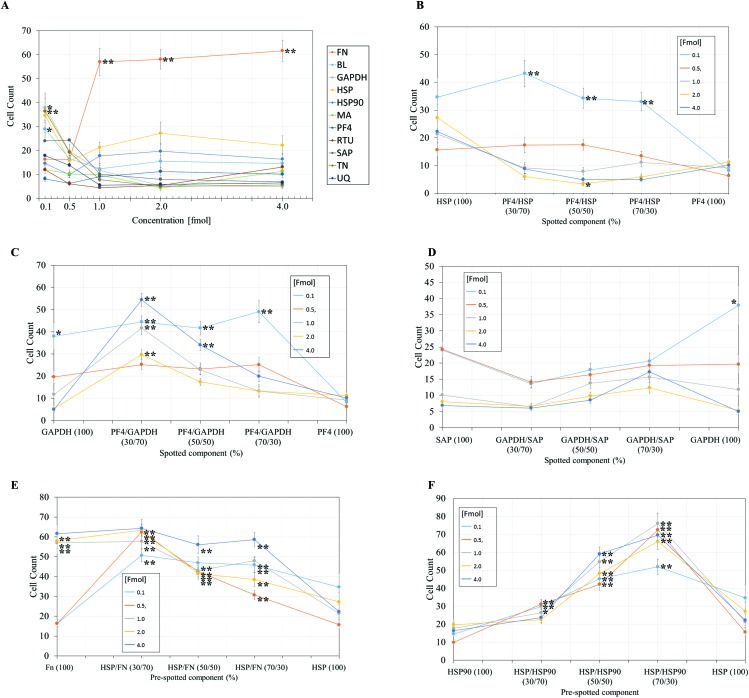
Pluripotent HUES7 cell count to protein adsorbed polyHPhMA surfaces at different fmol concentrations, data from secondary screen. *N* = 28 for all samples. Significant results from non-pretreated polyHPhMA surfaces are depicted with asterisks, * *p* < 0.0004, ** *p* < 0.0001. (A) HUES7 cell adherence to single proteins spotted on polyHPhMA. For images B–F samples are spotted at different fmol concentrations, 0.1, 0.5, 1, 2, 4 fmol. (B) HUES7 cell adherence to heat shock protein and platelet factor 4 protein single and co-adsorbed polyHPhMA spots. (C) HUES7 cell adherence to GAPDH and platelet factor 4 protein single and co-adsorbed polyHPhMA spots. (D) HUES7 cell adherence to GAPDH and serum amyloid-P component protein single and co-adsorbed polyHPhMA spots. (E) HUES7 cell adherence to fibronectin and heat shock protein single and co-adsorbed polyHPhMA spots. (F) HUES7 cell adherence to heat shock protein 90 and heat shock protein single and co-adsorbed polyHPhMA spots. Error bars are standard error of the mean, *n* = 28 on one array, raw data in Fig. 2B. BL = Beta-lactoglobulin, TN = tetranectin, PF4 = platelet factor 4, GAPDH = glyceraldehyde-3-phosphate dehydrogenase, MA = agrin, UQ = ubiquitin, HSP90 = heat shock protein 90, HSP = heat shock protein-1-like, SAP = serum amyloid P, FN = fibronectin and RTU = reticulon 4 interacting protein 1.

**Fig. 5:**
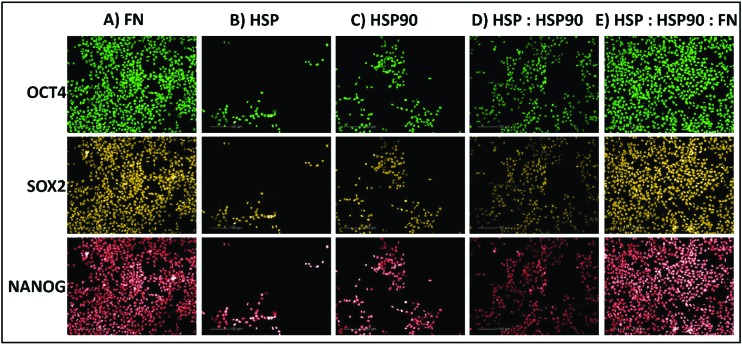
Immunocytochemistry staining for positive pluripotent marker (OCT4, SOX2 & NANOG) expression in hiPSCs (ReBl-PAT) after 3 days culture on protein adsorbed polyHPhMA surfaces. (A) FN = 100, (B) HSP = 100, (C) HSP90 = 100, (D) HSP : HSP90 = 70 : 30, (E) HSP : HSP90 : FN = 35 : 15 : 50.

Five protein combinations were also selected for further investigation as they were supportive of hESC attachment at the primary screen stage (HSP : HSP90, PF4 : HSP, PF4 : GAPDH, HSP : FN, and GAPDH : SAP). Thus, these combinations were evaluated further at a greater range of dosing compositions (30%, 50%, and 70%) and concentrations (0.1, 0.5, 1, 2, and 4 fmol) using the same HUES-7 cell conditions as before in the primary screen. To ensure confidence in the data, the secondary screen used 28 replicates per polymer pretreatment. Pluripotency was also assessed using a ReBl-PAT, a human induced pluripotent stem cell line (hiPSC), *via* OCT-4, NANOG and SOX-2 expression after 3 day culture on the best polyHPhMA protein pretreatments identified from Fig. 3 and 4 scaled up into well plates and presented in Fig. 5.

The secondary screen determined 42 pretreatments which supported greater HUES-7 cell adherence than the non-pretreated polymer spots (*p* < 0.0004, Fig. 3). A heat map representative of pluripotent HUES-7 cell count for the secondary screen is shown in Fig. 2A. The top row on the heat map shows the pluripotent cell adherence on the non-pretreated polyHPhMA spots (cell count at an average of 14 ± 2.4 cells, *n* = 35). ‘Blocks’ below this row are representative of cell adherence to protein pretreated spots of polyHPhMA. The number of cells attached to each spot varied from very few (less than 10 cells per spot for PF4 samples at 0.1, 0.5 and 1 fmol) to greater than 60 cells per spot for HSP : HSP90 co-adsorbed at 70 : 30 for 0.5, 1, 2 and 4 fmol.

Fibronectin (FN) pretreated surfaces when spotted at 1, 2 and 4 fmol displayed high cell adherence (average of 57 ± 5.7, 58 ± 4.1, and 62 ± 4.5). In contrast, GAPDH, SAP, TN, UQ and PF4 : HSP pretreatments displayed low cell adherence at 1, 2 and 4 fmol (average cell counts <14 cells). In some instances cell adherence was unaffected by spotting concentration. HSP : FN and HSP : HSP90 pretreated polyHPhMA surfaces displayed high cell adherence compared to the non-pretreated surface across all concentrations (average cell numbers ranging from 23 to 76). In contrast, human agrin pretreatment displayed low cell adherence across all concentrations (average cell counts ranging from 4.4 to 12.0).

In the case of single protein pretreatment to polyHPhMA surfaces the number of cells per spot was sensitive to the identity of the proteins adsorbed on the surface (Fig. 4A). For protein combinations, cell adherence was not typically linearly correlated with protein amount such that in most instances an optimum ratio of spotted components could be found (Fig. 4B–F). Using the results from Fig. 4, we are able to hypothesise how the different protein pretreatments facilitated HUES-7 cell attachment, and why certain protein combinations had a synergistic effect in terms of HUES-7 cell adherence.

## Discussion

### Cell adherence on FN pretreated polymer spots

The HUES-7 cell adherence to 1, 2, and 4 fmol fibronectin (FN) adsorbed to polyHPhMA spots was significantly higher (*p* < 0.0001), unpaired *t*-test than the non-pretreated spots with cells per spot as high as 110 cells per spot (Fig. 2). FN is an ECM protein widely reported to support pluripotent stem cell adherence when adsorbed to surfaces.^17^ Cell adherence to FN adsorbed surfaces was also seen to be dependent on the concentration of the spotted solutions with insignificant cell adherence at 0.1 and 0.5 fmol, but increasing markedly and plateauing at 1 fmol, comparable to similar experiments conducted by other groups (Fig. 4A).^18^ We hypothesize that FN either desorbs during culture from surfaces spotted at the higher concentrations prior to cell attachment, thus leaving the same protein density on the surface as found from the lower concentration drops resulting in comparable cell adherence on these samples, or that a saturation of surface ligands is reached at the 1 fmol drop concentration. Pluripotency was also assessed using a second cell line (ReBl-PAT) *via* NANOG and SOX-2 expression after 3 day culture presented in Fig. 5.

### A putative mechanism for cell adherence on HSP/HSP90 adsorbed polyHPhMA surfaces

The results from HSP/HSP90 adsorbed polyHPhMA show levels of cell adherence comparable to that seen for FN pretreatment Fig. 4A and F). HUES-7 cell adherence to 0.5 and 1 fmol HSP : HSP90 spotted at a 70 : 30 ratio was significantly higher that fibronectin adsorbed spots (*p* < 0.05, unpaired *t*-test). The trend for cell adherence on HSP and HSP90 co-adsorbed surfaces was the same across all the concentrations used (Fig. 4F). A clear linear increase in cell adherence is shown as HSP is added to the spotting solutions (0, 30, 50 and 70%) for all concentrations. However, at 100% HSP, hESC adherence dropped notably. Longer term studies on a second cell line (ReBl-PAT) also demonstrated that cells maintained pluripotency (assessed *via* OCT-4, NANOG, and SOX-2 expression) on the HSP : HSP90 70 : 30 ratio in greater numbers than on each separate pretreated component (HSP *vs*. HSP90, Fig. 5). The trend suggests a synergistic effect between these two proteins, which is not entirely unexpected as they are both members of the heat shock protein family. HSP functions as a molecular ‘chaperone’ and aids in folding translated peptides and proteins (HSP is present in its native state in the cell cytoplasm/nucleus). HSP is from the HSP70 family of heat shock proteins which have been reported to have altered expression in mouse ESCs.^19^ Expression of HSPa1a and HSPa1b were observed to decrease in 4-day old embryoid bodies.^20^ HSP may also drive Nanog expression (a key regulatory protein in maintaining self-renewal of human pluripotent stem cells).^19,21,22^ Activation of transcription factor 3 (STAT3) drives upregulation of Nanog expression (observed in mouse PSCs only).^23–25^ A proposed mechanism is that HSP70 (along with other proteins) is involved in the phosphorylation and nuclear translocation of STAT3. While this mechanism appears to be only present for murine PSCs and not human, their remains the possibility of a similar pathway using a different transcription factor. HSP90 has been reported as a requirement for mouse ES cell pluripotency.^26^ In the same paper the authors noted a critical ‘chaperone’ role of HSP90. HSP90 physically associates with and thus prevents proteasomal degradation of Nanog and Oct-4. Notably HSP90 inhibition resulted in a drop in Oct-4 messenger ribonucleic acid (mRNA) in mouse and human ESCs as well as in embryoid bodies. HSP90 inhibition also resulted in increased expression of markers indicating early differentiation to a mesodermic state. HSP90 is believed to modulate the STAT3 pathway on mouse ESCs *via* the LIF receptor.^27^ HSP90 also forms chaperone complexes with HSP70 (*i.e.* the steroid aporeceptor complex). For the results shown here it is possible that both these proteins are internalised by HUES-7 cells and form chaperone complexes as well as maintain cell pluripotency. Heat shock proteins have been identified on MEF-CM pretreated Matrigel and gelatin surfaces among hundreds of other proteins adsorbed from media to support stem cell culture.^11,12^ The mechanism for adherence to surfaces co-adsorbed with these heat shock proteins is not clear (these do not contain RGD or other binding domains) and merit future investigations as cells per spot on these surfaces were comparable to the results obtained from FN adsorbed surfaces.

### Cell adherence on FN/HSP adsorbed polyHPhMA surfaces

HUES-7 cell adherence to co-adsorbed polyHPhMA surfaces with FN and HSP were all significantly greater (*p* < 0.0001, unpaired *t*-test) than that seen in the control (Fig. 4E) and comparable to results obtained from FN only pretreatment on the surface. The results for surface co-adsorbed with solutions containing 0.1 and 0.5 fmol solution were notable as they are significantly greater than that seen for the control. FN as a single component at these concentrations did not support pluripotent stem cell adherence at significant levels above the control, suggesting an additive effect from HSP.

### Cell adherence for other protein pretreated polyHPhMA surfaces

Cell adherence to GAPDH, BL, and TN on 0.1 fmol adsorbed surfaces was significantly greater (*p* < 0.0004, unpaired *t*-test) than that of the control and dropped to non-significant adherence at higher concentrations (Fig. 4A). None of these proteins have been previously investigated as a possible protein pretreatment candidate for adherence and maintenance of hESCs. GAPDH has been reported to bind to the uPAR receptor, and may explain the significant cell adherence on GAPDH pretreated surfaces.^28^ TN binds to plasminogen and is found in the ECM, hence adherence to TN is not entirely unexpected.

For SAP/GAPDH co-adsorbed surfaces cell adherence was not statistically different (0.5 > *p* > 0.025 unpaired *t*-test) to that of the control Fig. 4D. SAP appears to have an inhibitory/antagonistic role to GAPDH (which supported cell adhesion at higher levels than the control as a single component at 0.1 fmol and when co-adsorbed with PF4 at 0.1 fmol). SAP has been identified as an agent which prevents fibrocyte differentiation.^29^

Cell adherence to single and co-adsorbed GAPDH and PF4 surfaces are shown in Fig. 4C. Two separate trends are seen depending on the concentration of the spotted proteins. For 0.1 and 0.5 fmol adsorbed samples a gradual increase in cell count is observed as PF4 amount increases (up to 70% whereby it drops significantly when PF4 amount increases to 100%). For surfaces adsorbed with proteins spotted at a concentration of 1, 2 and 4 fmol an entirely different trend is observed, cell adherence to 100% GAPDH was low to start with (non-significant to the control, 0.3 > *p* > 0.0012, unpaired *t*-test) and rises sharply at a 70 : 30 ratio (GAPDH : PF4). As the PF4 amount increased cell numbers dropped linearly to statistically insignificant levels. The trend observed for the 0.1 and 0.5 fmol samples may represent the optimum packing density of GAPDH on the surface, as cells failed to adhere to surfaces adsorbed with high concentrations of GAPDH. PF4 appears to have an additive effect as the co-adsorbed samples outperform GAPDH-only samples in terms of cell count. For the 1, 2, and 4 fmol the trend is suggestive that cell interactions with PF4 are dominant.

Cell adherence to single and co-adsorbed HSP and PF4 surfaces are shown in Fig. 4B. While the results were largely non-significant relative to the control and cell adherence was minimal, at the 2 fmol 50 : 50 ratio average cell adherence is 3.4 cells per spot (*p* < 0.0004). This result suggests that significant amounts of adsorbed proteins are retained on the surface during culture, and the HSP/PF4 pretreatment discourages hESC adherence and expansion.

### Cell adherence on scaled protein pretreated polyHPhMA surfaces

We next sought to explore whether protein adsorbed polyHPhMA surfaces could support pluripotent stem cell culture in a scaled up well plate format. Cell adherence was supported on HSP, HSP90 and FN adsorbed surfaces, with pluripotent marker expression of OCT4, SOX2, and NANOG maintained following three days culture (all >95%) (Fig. 5). Scaled up culture confirmed that HSP and HSP90 could support pluripotent culture (HSP = 43 × 10^4^ ± 14.1 × 10^4^, HSP90 = 56.6 × 10^4^ ± 13 × 10^4^, *n* = 3) and the enhanced effect on cell number of co-adsorbed surfaces was still observed (HSP : HSP90 = 122.3 × 10^4^ ± 12.6 × 10^4^, *n* = 3). The effect of the addition of fibronectin to HSP : HSP90 pretreatment was also investigated and demonstrated no enhancement in cell number over fibronectin pretreatment alone in this scaled up format (FN = 189.6 × 10^4^ ± 8.5 × 10^4^, HSP : HSP90 : FN = 183 × 10^4^ ± 9.1 × 10^4^, *n* = 3).

## Conclusions

Using an analysis of proteins rinsed from a polystyrene derived surface supportive of pluripotent stem cell expansion we have identified the adsorbed proteins implicated in the maintenance of hESC pluripotency when culture is done in MEF-CM, a complex medium which was found to deposit both mouse and human-derived proteins onto the surface.

The development of a method to screen the effect of the material–protein biointerface on hESC adherence has allowed a detailed and simultaneous investigation of different protein pre-treatments (and substrates). Using the leads from the proteomics study, we have illustrated the technical feasibility of using robotic spotters to create a multitude of protein–material interfaces in high throughput.

The results from this approach indicate that HSP/HSP90 protein pretreatment encourages pluripotent cell attachment function on polyHPhMA. This has potential to provide information on the mechanism hESC response to man-made materials in complex medium. The identification of both these heat shock proteins as supportive of pluripotent HUES-7 hESC adherence and expansion has not been reported before. We postulate a mechanism based on the observation that cell response appears dependent on a synergistic effect of HSP with HSP90. These are new candidate proteins of importance in mediating pluripotent stem cell attachment to surfaces, similar to hESC adherence on previously reported proteins.^30^

## Experimental

### Proteomics reagents

Ultrapure (18.2 MΩ cm) H_2_O was from a MilliQ water purifier (Millipore, Billerica, USA, ; http://www.emdmillipore.com). All solvents (HPLC grade), and reagents were purchased from Sigma-Aldrich (Dorset, U.K, ; http://www.sigmaaldrich.com).

### Plasm etching of TCPS surfaces

Plasma etching of TCPS surfaces were achieved as described previously.^1^ Briefly, TCPS six-well plates (Sarstedt AG & Co, Numbrecht, Germany, ; http://www.sarstedt.com; and Nunc, Roskilde, Denmark, ; http://www.nuncbrand.com) were placed in a vacuum chamber (<20 mT) with oxygen gas flowing through at a pressure of 300 mT. A radio frequency source (power 20 W) was used to create plasma in the gas, and the TCPS plates were exposed to the plasma for 5 min and vented with nitrogen afterwards. The water contact angles for the TCPS before and after plasma etching treatment was determined with a CAM 200 Optical Contact Angle Meter (KSV Instruments Ltd, Helsinki, Finland, ; http://www.ksvltd.com).

### Surface pre-adsorption with MEF-CM

MEF-CM was prepared as described elsewhere.^31^ Briefly, MEFs (strain CD1, 13.5 days post coitum) were mitotically inactivated with mitomycin C (10 μg mL^–1^, 2.5 h) and seeded at 6 × 10^4^ cells per cm^2^ in T75 flasks. The next day, inactivated MEFs were washed with PBS and incubated with 25 mL of unconditioned medium (UM) for 24 h, at which time CM was collected for preadsorption study. Unconditioned medium composition consisted of DMEM-F12 (Thermo Fisher) supplemented with 15% KnockOut Serum Replacement (Invitrogen), 100 mM β-mercaptoethanol (Sigma), 1% non-essential amino acids (NEAA) (Thermo Fisher), 2 mM GlutaMAX (Thermo Fisher), and 4 ng mL^–1^ bFGF (R & D Systems). PE and non-PE treated Nuclon Delta 4-well plates (Nunc) were incubated with 5 mL MEF-CM per well at 37 °C for 1 hour prior to extraction of surface-bound proteins.

### Surface extraction of proteins

Conditioned medium pre-adsorbed PE-TCPS plates were washed with 2 mL of phosphate buffered saline (PBS) solution for 10 minutes while shaking a total of five times. Surface extraction of proteins was made with an extraction solvent made up of 1 M NaCl, 8 M urea, 1% Triton X (TX)-100, and 50% isopropanol. 100 μl of the extraction solvent was added to each of the wells of the six-well PE-TCPS and shaken for 30 min at room temperature. The extracted solutions were then desalted using acetone precipitation. 4× volume of cold acetone (–20 °C) was added to the extracted samples and incubated for 60 min at (–20 °C), which was the centrifuged at 13 000*g* for 10 min. The acetone was then removed and the protein pellet was resuspended in PBS. Extracted proteins from 12 wells were used for the subsequent proteomics analysis.

### Gel and staining

Samples from the extraction were run on a 4–12% bis–tris using MES buffer (Invitrogen, Paisley, UK) for 60 minutes at 200 V as per manufacturer's instructions. Silver staining was performed and Coomassie brilliant blue G-250 (Sigma-Aldrich, Dorset, U.K, http://www.sigmaaldrich.com) staining were performed as per previous instructions.^32,33^

### Proteomics analysis

1D gel bands were excised and transferred into a 96-well PCR plate. The gel bands were cut into 1 mm^2^ pieces, destained, reduced (dithiothreitol) and alkylated (iodoacetamide) and subjected to enzymatic digestion with trypsin overnight at 37 °C. Each gel lane was divided into 16 bands and digested separately. After digestion, the supernatants for two adjacent were pooled to give a total of 8 samples per lane.

All LC-MS/MS experiments were performed using a Dionex Ultimate 3000 RSLC nanoUPLC (Thermo Fisher Scientific Inc., Waltham, MA, USA) system and a Q-Exactive Orbitrap mass spectrometer (Thermo Fisher Scientific Inc., Waltham, MA, USA). Separation of peptides was performed by reversed-phase chromatography at a flow rate of 300 nL min^–1^ and a Thermo Scientific reversed-phase nano Easy-spray column (Thermo Scientific PepMap C18, 2 μm particle size, 100 Å pore size, 75 μm i.d. × 50 mm length). Solvent A was water + 0.1% formic acid and solvent B was 80% acetonitrile, 20% water + 0.1% formic acid. The linear gradient employed was 2–40% B in 30 min (total run time including high organic wash and re-equilibration was 60 min).

All *m*/*z* values of eluting ions were measured in an Orbitrap mass analyzer, set at a resolution of 70 000. Data dependent scans (top 20) were employed to automatically isolate and generate fragment ions by higher energy collisional dissociation (HCD) in the quadrupole mass analyser and measurement of the resulting fragment ions was performed in the Orbitrap analyser, set at a resolution of 17 500. Peptide ions with charge states of 2+ and above were selected for fragmentation.

Post-run, the data was processed using Protein Discoverer (version 1.4., ThermoFisher). MS/MS data were then submitted to the Mascot search algorithm (Matrix Science, London UK) and searched against the Uniprot mouse database (UniProt_Mouse_Jan12 mouse_2012_01, 85 691 sequences; 38 653 293 residues) using a fixed modification of carbamidomethyl (C) and variable modifications of oxidation (M) and deamidation (NQ). Peptide identifications were accepted if they could be established at greater than 95.0% probability.

### Contact printing

Polymer microarrays were made using published protocols.^34,35^ A 6% (weight/volume, w/v) pHEMA solution was made by dissolving 3 g of pHEMA in 50 mL of an ethanol/water mixture (95 : 5). Epoxy-coated slides (Genetix, San Jose, California, USA) were dip-coated in the pHEMA solution and dried for 2 hours. The dip coated slides were then left for a period of at least a week to allow for evaporation of the solvent. Monomer solutions of the acrylamide monomer were prepared at a ratio of 25% (v/v) monomer, 75% (v/v) DMF and 1% (w/v) 2,2-dimethoxy-2-phenylacetope-none. These solutions were prepared in a 384-well polypropylene plate (20 μl volumes).

Prior to printing the pins and pin holder are cleaned by sonication for 10 min in dichloromethane. Array fabrication was designed *via* the in-built software. Printing was then carried out using 946MP6B slit pins (these pins have a diameter of 220 μm, Arrayit) on a BioDot contact printer at 30% humidity at room temperature. The array fabrication took place in an argon atmosphere, the oxygen level was reduced to less than 2000 parts per million (ppm). The Biodot robot prints polymer spots using an *x*, *y*, *z* stage. The array dimensions were 38 spots in *x* by 105 spots in *y* on a pHEMA coated epoxy glass slide, polymer spots were 300 μm in diameter. Spot to spot distance was 500 μm. After printing slides were left for at least a week in high vacuum conditions to allow for DMF extraction.

### Protein printing

Proteins were spotted using a Scienon S11 piezo spotter. Proteins were prepared individually in a 384 well plate and co-adsorbed solutions were prepared by mixing on polymer spots. Printing took place at near dew point conditions by lowering the stage temperature to 8 °C, and set humidity at 40% to elongate the sessile drop presence. To prevent protein precipitation from drying, hydration of the protein solutions was achieved with dispensed water drops. In this manner protein array fabrication could take hours without protein spots drying. At the end of the protein printing, slides were immersed into deionised water while sessile protein drops were still present on the surface of the polymer spots. For the preliminary array print, proteins were printed at either 0.1 fmol, 0.5 fmol, or 1 fmol. Binary mixtures were also prepared pairwise for 169 unique combinations (in a 70 : 30 ratio) and printed at the three concentrations. A 70 : 30 ratio was used to assess in parallel cell adherence to biomolecules when spotted as a minor or major component. Using PDCs on slide mixing was achieved, 7 drops of one protein and 3 drops of another protein were spotted and mixed on surface to make mixed protein solutions. Due to an error in printing alpha-2-antiplasmin (A2A), thirty-nine samples were unsuitable for screening. A secondary screening of protein ‘hits’ was fabricated using a refined set of protein pretreatments from the primary screen. Proteins were printed at 0.1, 0.5, 1, 2 and 4 fmol. Selected protein mixtures were also prepared at 3 ratios 30 : 70, 50 : 50 and 70 : 30 and printed at the five concentrations. Using PDCs on slide mixing was achieved, 7 drops of one protein and 3 drops of another protein were spotted and mixed on surface to make mixed protein solutions of a 70 : 30 and 30 : 70 ratio. To achieve the 50 : 50 ratio 5 drops of each protein were mixed in the same manner. In this manner, 131 unique surfaces were investigated.

### Cell seeding and analysis of microarray

HUES-7 cells were seeded at a density of 1 × 10^6^ cells in StemPro, 1% w/v antibiotic (penicillin/streptomycin). After seeding cell nuclei were stained using DAPI. To assess cell pluripotency OCT-4 expression was detected. Arrays were imaged using an Ix51 IMSTAR microscope in brightfield. Arrays were also imaged using a DAPI and Cy3 filter. All images were captured using a ProgRes MF (Jenoptik) monochrome CCD digital camera. Cell counting was achieved using CellProfiler. Only pluripotent cells were counted using images acquired from the Cy3 filter.

### Statistics

Analysis of pluripotent cells per spot was carried out using statistical significance using an unpaired *T*-test method and correcting using the Bonferroni correction method. Statistical significance is usually set at *p* < 0.05, however it is advised to take into account multiple comparisons when analysing large datasets. The Bonferroni correction divides 0.05 by the number of unique samples (not replicates). Using this method statistical significance from the control was found to be p < 0.0001 for the primary screen ND *P* < 0.0004 for the secondary screen. The control was cell number on the non-pretreated *N*-(4-hydroxyphenyl) methacrylamide spots.

### Polymer scale up

To allow longer term culture and assessment of pluripotency, TCPS six-well plates were oxygen plasma etched in a Si-free reactor at 100 W for 10 minutes prior to coating with polymer solution. Prepolymerised polymer was dissolved in ethanol to make a 5% w/v solution. 100 μL of polymer solution was pipetted into each well, the plate was loosely closed and allowed to dry overnight at ambient conditions. Polymer coated plates were exposed to 1 ml of protein mixture for 1 hour at 37 °C to allow protein adsorption to occur, followed by PBS washing to remove unbound protein. 0.5 × 10^6^ ReBl-PAT pluripotent stem cells were seeded into each well in StemPro, 1% w/v antibiotic (penicillin/streptomycin). Pluripotency was assessed following three days culture with daily media exchanges by immunocytochemistry staining for positive OCT4, SOX2 & NANOG expression.
