# Supplementary material for: Identification of polymer surface adsorbed proteins implicated in pluripotent human embryonic stem cell expansion
Source: Biomater Sci. 2016 Jul 28;4(9):1381–91. doi: 10.1039/c6bm00214e (PMC5038343; doi:10.1039/c6bm00214e)
Supplement: Supplementary file 1 [file BM-004-C6BM00214E-s001.pdf]

Electronic Supplementary Material (ESI) for Biomaterials Science.
This journal is © The Royal Society of Chemistry 2016

1. Proteomics

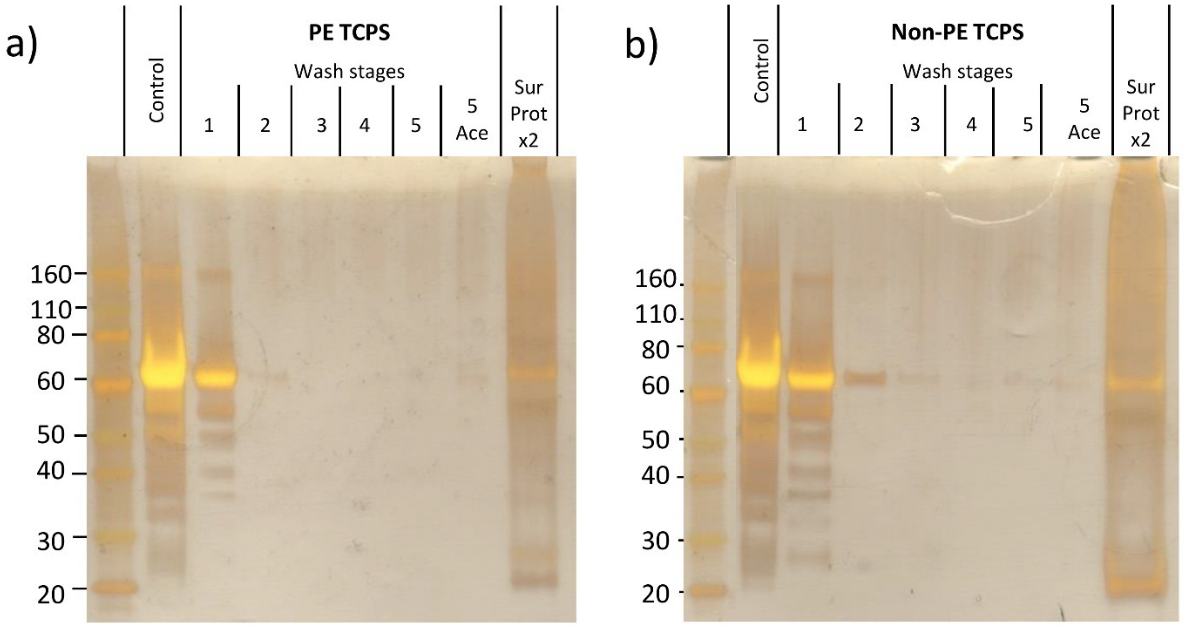

Figure 1 In order to analyse adsorbed proteins from the surface of TCPS plates it was necessary to remove all soluble proteins that may have been left behind from the MEF medium and it was found that a wash procedure consisting of five PBS rinses was sufficient to remove the majority of soluble proteins from the surface. The optimal extraction solvent was determined to be 1M NaCl, 8M urea, 1% TX-100 and 50% isopropanol, which was found to be sufficient to remove surface bound proteins from PE (A) and non-PE (B) treated TCPS plates.

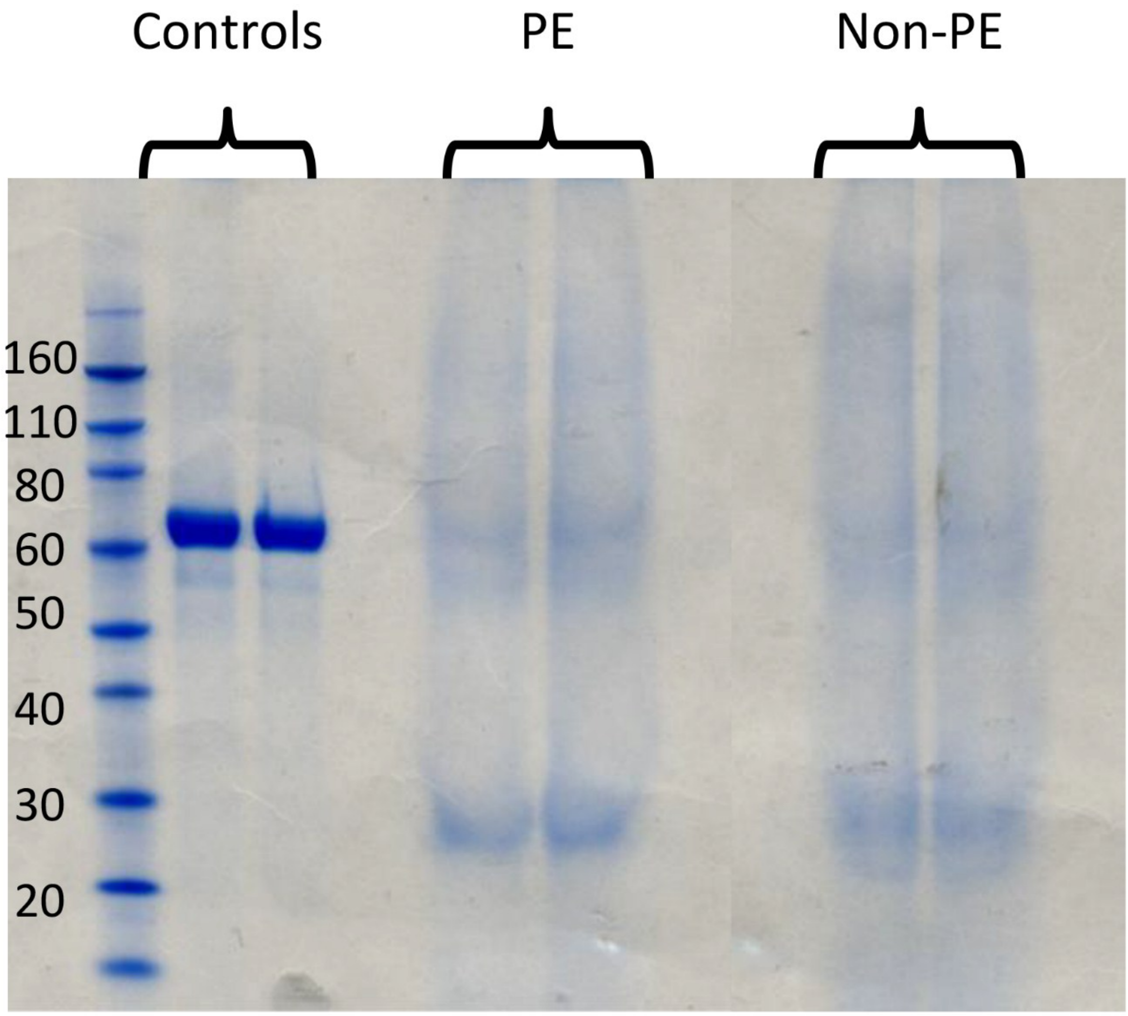

Figure 2 An intensity of the proteins from 12 wells was found to be needed for proteomics analysis, which could be observed using standard Coomassie staining.

Biomolecule	Molecular weight	Concentration when printing at 1 fmol	Source
Platelet factor 4	29 kDa	8.055 µg/mL	Abcam (ab80477)
Tetranectin	25 kDa	6.94 µg/mL	R&D Systems (5170-CL-050)
Serum amyloid P-component	786 kDa	218 µg/mL	Anaspec (62922)
Beta-lactoglobulin	19,883 Da	5.522 µg/mL	Mybiosource.com (MBS717034)
Alpha-2-antiplasmin	70,000 Da	19.44 µg/mL	Abcam (ab77936)
Fibronectin	440 kDa	125 µg/mL	Sigma Aldrich (F2006)
Desmoplakin	331,774 Da	92.16 µg/mL	Abcam (ab71689)
Heat shock protein 1-like	70.46 kDa	19.57 µg/mL	Abnova (H00003305-T01)
Glyceraldehyde-3-phosphate dehydrogenase	36 kDa	10 µg/mL	Mybiosource.com (MBS203254)
Reticulon 4 interacting protein 1	43.6 kDa	12.11 µg/mL	Abnova (H00084816-T02)
Agrin	110 kDa	30.56 µg/mL	R&D Systems (6624-AG-050)
Ubiqutin	8565 Da	2.38 µg/mL	Abcam (ab51097)
Heat shock protein 90	86.8 kDa	24.11 µg/mL	Mybiosource.com (MBS203032)

Table 1 Identity of proteins and concentrations used when spotting at 1 fmol.

Table 1 displays the biomolecules used in this experiment Proteins used differ from those identified by the proteomic work. Human proteins were used where possible with the exception of beta-lactoglobulin (bovine). Human desmoplakin I + II peptide was also used instead of desmoplakin. Reticulon 4 interacting protein 1 was used in place of reticulon 4 protein. Mini-agrin is a murine protein and therefore human agrin was used as an alternative. Heat shock protein 90 was also used in place of heat shock protein 90 beta member 1. Proteomics analysis also identified ubiquitin subunit 1, but full length ubiquitin was used instead.

2. Primary screen Print

BL/A2A	DPK/A2A	FN/A2A	GAPDH/A2A	GAPDH/A2A	HSP90/A2A	MA/A2A	PF4/A2A	RTU/A2A	SAP/A2A	TN/A2A	UQ/A2A
BL	DPK/BL	FN/BL	GAPDH/BL	GAPDH/BL	HSP90/BL	MA/BL	PF4/BL	RTU/BL	SAP/BL	TN/BL	UQ/BL
BL/DPK	DPK	FN/DPK	GAPDH/DPK	GAPDH/DPK	HSP90/DPK	MA/DPK	PF4/DPK	RTU/DPK	SAP/DPK	TN/DPK	UQ/DPK
BL/FN	DPK/FN	FN	GAPDH/FN	GAPDH/FN	HSP90/FN	MA/FN	PF4/FN	RTU/FN	SAP/FN	TN/FN	UQ/FN
BL/GAPDH	BL/GAPDH	FN/GAPDH	GAPDH	HSP/GAPDH	HSP90/GAPDH	MA/GAPDH	PF4/GAPDH	RTU/GAPDH	SAP/GAPDH	TN/GAPDH	UQ/GAPDH
BL/HSP	DPK/HSP	FN/HSP	GAPDH/HSP	HSP	HSP90/HSP	MA/HSP	PF4/HSP	RTU/HSP	SAP/HSP	TN/HSP	UQ/HSP
BL/HSP90	DPK/HSP90	FN/HSP90	GAPDH/HSP90	GAPDH/HSP90	HSP90	MA/HSP90	PF4/HSP90	RTU/HSP90	SAP/HSP90	TN/HSP90	UQ/HSP90
BL/MA	DPK/MA	FN/MA	GAPDH/MA	GAPDH/MA	HSP90/MA	MA	PF4/MA	RTU/MA	SAP/MA	TN/MA	UQ/MA
BL/PF4	DPK/PF4	FN/PF4	GAPDH/PF4	GAPDH/PF4	HSP90/PF4	MA/PF4	PF4	RTU/PF4	SAP/PF4	TN/PF4	UQ/PF4
BL/RTU	DPK/RTU	FN/RTU	GAPDH/RTU	GAPDH/RTU	HSP90/RTU	MA/RTU	PF4/RTU	RTU	SAP/RTU	TN/RTU	UQ/RTU
BL/SAP	DPK/SAP	FN/SAP	GAPDH/SAP	GAPDH/SAP	HSP90/SAP	MA/SAP	PF4/SAP	RTU/SAP	SAP	TN/SAP	UQ/SAP
BL/TN	DPK/TN	FN/TN	GAPDH/TN	GAPDH/TN	HSP90/TN	MA/TN	PF4/TN	RTU/TN	SAP/TN	TN	UQ/TN
BL/UQ	DPK/UQ	FN/UQ	GAPDH/UQ	GAPDH/UQ	HSP90/UQ	MA/UQ	PF4/UQ	RTU/UQ	SAP/UQ	TN/UQ	UQ

Table 2 Layout of array plan, co-adsorbed mixtures were prepared by spotting 3 drops of one protein followed by spotting 7 drops of another protein. Each cell is 1 x 7 polymer spots. Two further arrays were printed on the same slide as proteins were spotted at 3 different concentrations. BL = Beta-lactoglobulin, A2A = alpha-2-antiplasmin, TN = tetranectin, PF4 = platelet factor 4, GAPDH = glyceraldehyde–3-phosphate dehydrogenase, MA = agrin, UQ = ubiquitin, HSP90 = heat shock protein 90, DPK = desmoplakin, HSP = heat shock protein-1-like, SAP = serum amyloid P, Fn = fibronectin and RTU = reticulon 4 interacting protein 1

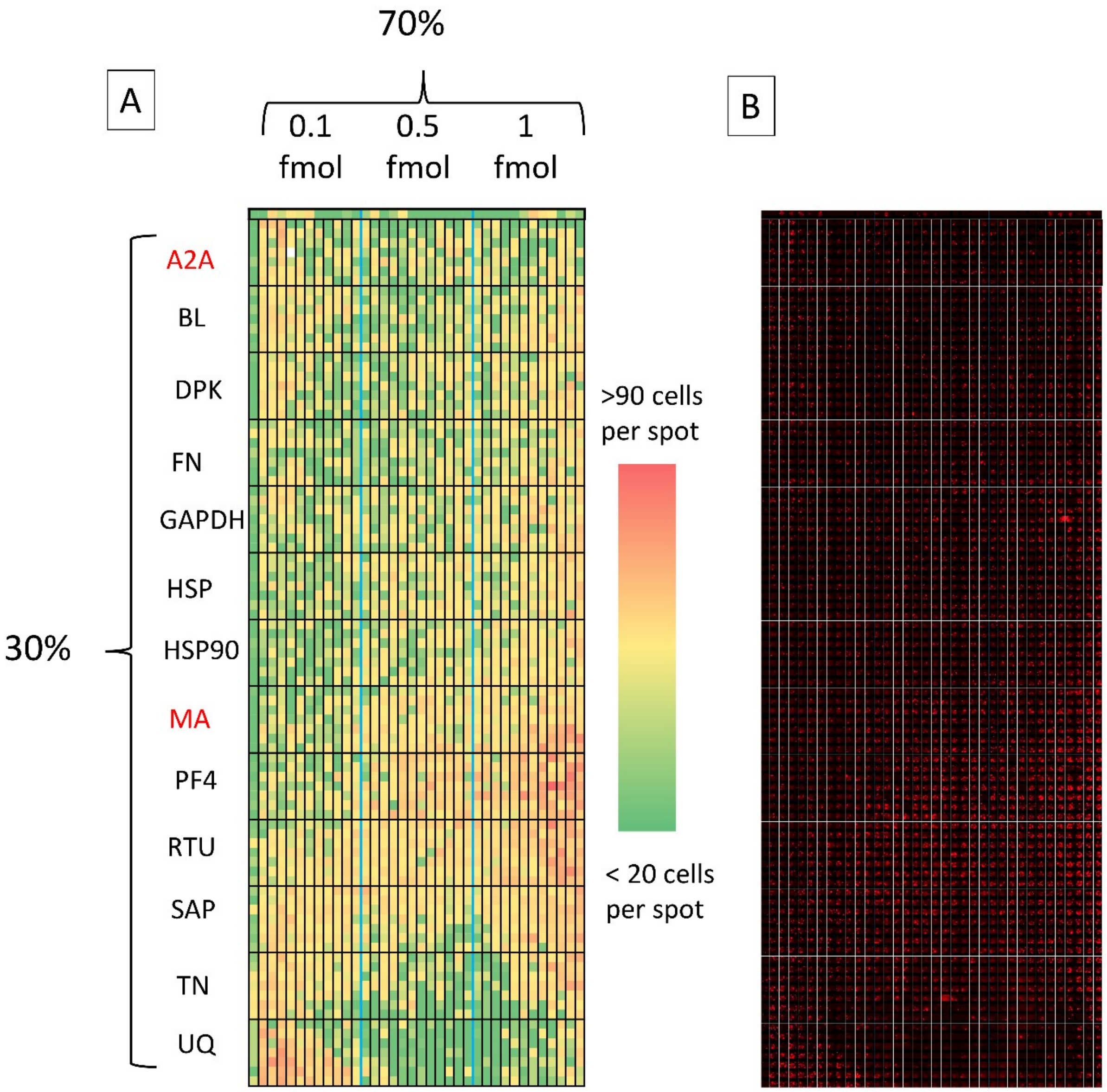

Figure 3 **A**) Collated images of the preliminary screen array. From left to right: heat map representative of cell attachment is shown (red signifies > 90 cells per spot, green signifies < 20 cells per spot). Each spot acquired separately and stitched together. 'Blocks' are separated by light blue lines, the 'block' on the left signifies cell attachment on surfaces spotted with 0.1 fmol protein, the centre 'block' shows cell attachment on surfaces spotted with 0.5 fmol, and the 'block' on the right shows cell adherence on surfaces spotted with 1 fmol. Columns (signified by bold black lines) represent different proteins spotted at 7 drops (from left to right these are BL, DPK, FN, GAPDH, HSP, HSP90, MA, PF4, RTU, SAP, TN and UQ). A2A columns did not print at all due to a blocked PDC nozzle head. Rows are separated by horizontal black lines and the order is shown above. MA did not spot due to a blocked PDC nozzle. The top row depicts cell adherence on non-spotted N-(4-Hydroxyphenyl) methacrylamide polymer spots. **B**) Detection of Oct-4 expressing cells on the array using Cy3 labelled antibody

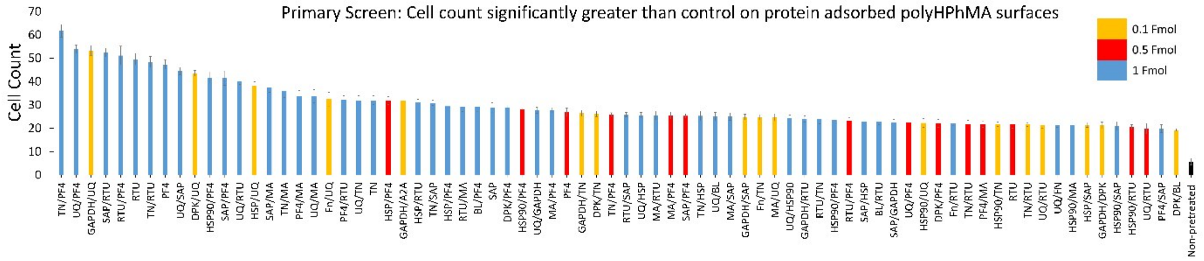

Figure 4 HUES7 cell count to protein adsorbed surfaces, only samples with cell adhesion significantly greater than control ($p < 0.0004$) are shown. Error bars are standard error of mean. HUES7 cell adherence to samples from preliminary screening array, n=7 from one microarray, raw data in Figure 2B. Bars are colour coded to represent concentration of spotted proteins. Yellow: 0.1 fmol, red: 0.5 fmol, and cyan: 1 fmol. The non-pretreated surface is shown in black.

3. **Secondary screen Print**

The ratios were used to assess in parallel the effect of cell adherence to biomolecules spotted as a major, minor or equivalent component on the surface.

	0.1 fmol	0.5 fmol	1 fmol	2 fmol	4 fmol
Ratio%	Bare	Bare	Bare	Bare	Bare
100	BL	BL	BL	BL	BL
100	Fn	Fn	Fn	Fn	Fn
100	GAPDH	GAPDH	GAPDH	GAPDH	GAPDH
100	HSP	HSP	HSP	HSP	HSP
100	HSP90	HSP90	HSP90	HSP90	HSP90
100	MA	MA	MA	MA	MA
100	PF4	PF5	PF6	PF7	PF8
100	RTU	RTU	RTU	RTU	RTU
100	SAP	SAP	SAP	SAP	SAP
100	TN	TN	TN	TN	TN
100	UQ	UQ	UQ	UQ	UQ
30/70	GAPDH/SAP	GAPDH/SAP	GAPDH/SAP	GAPDH/SAP	GAPDH/SAP
50/50	GAPDH/SAP	GAPDH/SAP	GAPDH/SAP	GAPDH/SAP	GAPDH/SAP
70/30	GAPDH/SAP	GAPDH/SAP	GAPDH/SAP	GAPDH/SAP	GAPDH/SAP
30/70	HSP/HSP90	HSP/HSP90	HSP/HSP90	HSP/HSP90	HSP/HSP90
50/50	HSP/HSP90	HSP/HSP90	HSP/HSP90	HSP/HSP90	HSP/HSP90
70/30	HSP/HSP90	HSP/HSP90	HSP/HSP90	HSP/HSP90	HSP/HSP90
30/70	HSP/FN	HSP/FN	HSP/FN	HSP/FN	HSP/FN
50/50	HSP/FN	HSP/FN	HSP/FN	HSP/FN	HSP/FN
70/30	HSP/FN	HSP/FN	HSP/FN	HSP/FN	HSP/FN
30/70	PF4/GAPDH	PF4/GAPDH	PF4/GAPDH	PF4/GAPDH	PF4/GAPDH
50/50	PF4/GAPDH	PF4/GAPDH	PF4/GAPDH	PF4/GAPDH	PF4/GAPDH
70/30	PF4/GAPDH	PF4/GAPDH	PF4/GAPDH	PF4/GAPDH	PF4/GAPDH
30/70	PF4/HSP	PF4/HSP	PF4/HSP	PF4/HSP	PF4/HSP
50/50	PF4/HSP	PF4/HSP	PF4/HSP	PF4/HSP	PF4/HSP
70/30	PF4/HSP	PF4/HSP	PF4/HSP	PF4/HSP	PF4/HSP

Table 3 Secondary array plan. Cells with 'Bare' represent 7 x 1 spots. All other cells represent 7 x 4 spots (making for 28 replicates per co-adsorbed sample). BL = Beta-lactoglobulin, TN = tetranectin, PF4 = platelet factor 4, GAPDH = glyceraldehyde–3-phosphate dehydrogenase, MA = agrin, UQ = ubiquitin, HSP90 = heat shock protein 90, HSP = heat shock protein-1-like, SAP = serum amyloid P, Fn = fibronectin and RTU = reticulon 4 interacting protein 1
